# Application of machine learning missing data imputation techniques in clinical decision making: taking the discharge assessment of patients with spontaneous supratentorial intracerebral hemorrhage as an example

**DOI:** 10.1186/s12911-022-01752-6

**Published:** 2022-01-13

**Authors:** Huimin Wang, Jianxiang Tang, Mengyao Wu, Xiaoyu Wang, Tao Zhang

**Affiliations:** 1 Department of Epidemiology and Health Statistics, West China School of Public Health and West China Fourth Hospital, Sichuan University, Chengdu, 610041 Sichuan China; 2 Department of Neurosurgery, West China Hospital, Sichuan University, Chengdu, 610044 Sichuan China

**Keywords:** Clinical decision making, Missing data, Imputation, Machine learning, Ensemble learning, Discharge assessment, Spontaneous supratentorial intracerebral hemorrhage

## Abstract

**Background:**

There are often many missing values in medical data, which directly affect the accuracy of clinical decision making. Discharge assessment is an important part of clinical decision making. Taking the discharge assessment of patients with spontaneous supratentorial intracerebral hemorrhage as an example, this study adopted the missing data processing evaluation criteria more suitable for clinical decision making, aiming at systematically exploring the performance and applicability of single machine learning algorithms and ensemble learning (EL) under different data missing scenarios, as well as whether they had more advantages than traditional methods, so as to provide basis and reference for the selection of suitable missing data processing method in practical clinical decision making.

**Methods:**

The whole process consisted of four main steps: (1) Based on the original complete data set, missing data was generated by simulation under different missing scenarios (missing mechanisms, missing proportions and ratios of missing proportions of each group). (2) Machine learning and traditional methods (eight methods in total) were applied to impute missing values. (3) The performances of imputation techniques were evaluated and compared by estimating the sensitivity, AUC and Kappa values of prediction models. (4) Statistical tests were used to evaluate whether the observed performance differences were statistically significant.

**Results:**

The performances of missing data processing methods were different to a certain extent in different missing scenarios. On the whole, machine learning had better imputation performance than traditional methods, especially in scenarios with high missing proportions. Compared with single machine learning algorithms, the performance of EL was more prominent, followed by neural networks. Meanwhile, EL was most suitable for missing imputation under MAR (the ratio of missing proportion 2:1) mechanism, and its average sensitivity, AUC and Kappa values reached 0.908, 0.924 and 0.596 respectively.

**Conclusions:**

In clinical decision making, the characteristics of missing data should be actively explored before formulating missing data processing strategies. The outstanding imputation performance of machine learning methods, especially EL, shed light on the development of missing data processing technology, and provided methodological support for clinical decision making in presence of incomplete data.

## Background

Medical data mainly comes from electronic medical records, medical images, etc. Due to factors such as difficulty in measuring some indicators, untimely data collection, improper data storage and difficulty in sharing medical information across platforms, there are often many missing values in medical data [1, 2], which directly affect clinical decision making such as disease diagnosis, treatment selection, discharge assessment and prognosis evaluation. Therefore, it is necessary to effectively process the missing data to improve the quality of medical data and the accuracy of clinical decision making.

Discharge assessment is an important part of clinical decision making. Whether the scientific and accurate discharge assessment can be made is not only related to the health outcomes, medical expenses and quality of life after discharge [3], but also closely related to the utilization efficiency of medical resources and the social medical burden [4, 5], which also puts forward high requirements for the quality of medical data.

To this end, this study took the discharge assessment of patients with spontaneous supratentorial intracerebral hemorrhage as an example to study the processing of missing data in clinical decision making. Spontaneous intracerebral hemorrhage is defined as intracerebral hemorrhage without trauma or surgery [6]. It is usually manifested as hematoma that expands in the brain parenchyma and may spread to the ventricular system and subarachnoid space or dural space [7]. Worldwide, although spontaneous intracerebral hemorrhage accounts for 15% of all stroke cases, it is associated with half of stroke-related deaths and 42% of stroke-related disability adjusted life-years lost [8]. Spontaneous supratentorial intracerebral hemorrhage is a kind of spontaneous intracerebral hemorrhage. It affects 4 million patients worldwide each year and median case fatality at 30-day is 40% [9, 10].

The volume of supratentorial hemorrhage is not only an important index for setting inclusion and exclusion criteria, comparing curative effects, predicting mortality and neurological prognosis [7, 11–14], but also widely used in clinical decision making. However, in practical work, the volume of hemorrhage is mostly calculated from the size of hematoma shown on CT [15], and the volume of hemorrhage in the ventricle is more difficult to be measured accurately. Clinically, more attention is paid to the size of hematoma, and the record of hemorrhage volume may be ignored. Moreover, the missing of the supratentorial hemorrhage volume is also the most obvious (11.65%) among all the variables in the data set used in this study.

A large number of important machine learning methods have emerged since the 1980s and 1990s, such as back propagation neural network and random forest (RF), which had a profound impact on the medical field including clinical decision making in presence of missing data. Before that, the traditional methods used to process the missing data in clinical decision making mainly included complete case analysis, mean imputation, k-nearest neighbors (KNN), expectation maximization and so on. With the in-depth application of machine learning models in this field, researchers found that machine learning models can restore the true distribution of data from missing data sets more accurately than the traditional missing data processing models. For example, Sun YV et al. used neural networks (NN) to impute real genotype data and found that when the proportion of missing data was 1%-5%, the imputation accuracy of NN was higher than that of the expectation maximization method [16]. Furthermore, some important previous studies, using cutting-edge technologies such as statistical simulation, found that the ensemble learning (EL) model can more accurately restore the real distribution of data than single learners [17, 18].

However, the previous model evaluations criteria were mainly based on whether the missing data processing model can restore the true distribution of data, mostly adopting the errors between the actual values and the imputation values as the evaluation metrics. But for real-world clinical decision making, those criteria may be too strict. Because the reasons for the data missing in the real world are very complicated, it is almost impossible to make the imputed data distribution completely consistent with the underlying true distribution. In contrast, from the actual needs of clinical decision making, even if there are some differences between the two distributions, as long as the differences do not affect the accuracy of decision-making results, it also has clinical values. Therefore, in order to meet the actual needs of clinical decision making for missing data processing, this study adjusted the evaluation criteria to transform the previous evaluation of the consistency of data distribution into the evaluation of the impact of clinical decision-making results.

To this end, using the missing data processing evaluation criteria more suitable for clinical decision making, this study aimed at systematically exploring the performance and applicability of several machine learning algorithms commonly used in current researches under different data missing scenarios, and whether these machine learning algorithms were more advantageous than traditional methods, in order to provide basis and reference for the selection of suitable missing data processing method in practical clinical decision making.

## Methods

### Data source and preprocessing

The data in this study came from the database of Comprehensive Data Collection and Decision Support System for health statistics in Sichuan Province. This database includes the medical records of patients with spontaneous intracerebral hemorrhage in all general hospitals and community hospitals in Sichuan Province since January 1, 2017. In order to better explain the research problems and operability, the medical records of 2000 patients with spontaneous intracerebral hemorrhage who were admitted to the hospital until June 30, 2019 were randomly selected, and the cases with missing value were excluded. At the same time, the patients with supratentorial hemorrhage were selected as the research objects, and finally 1468 complete samples were included.

### Experimental design

**Fig. 1:**
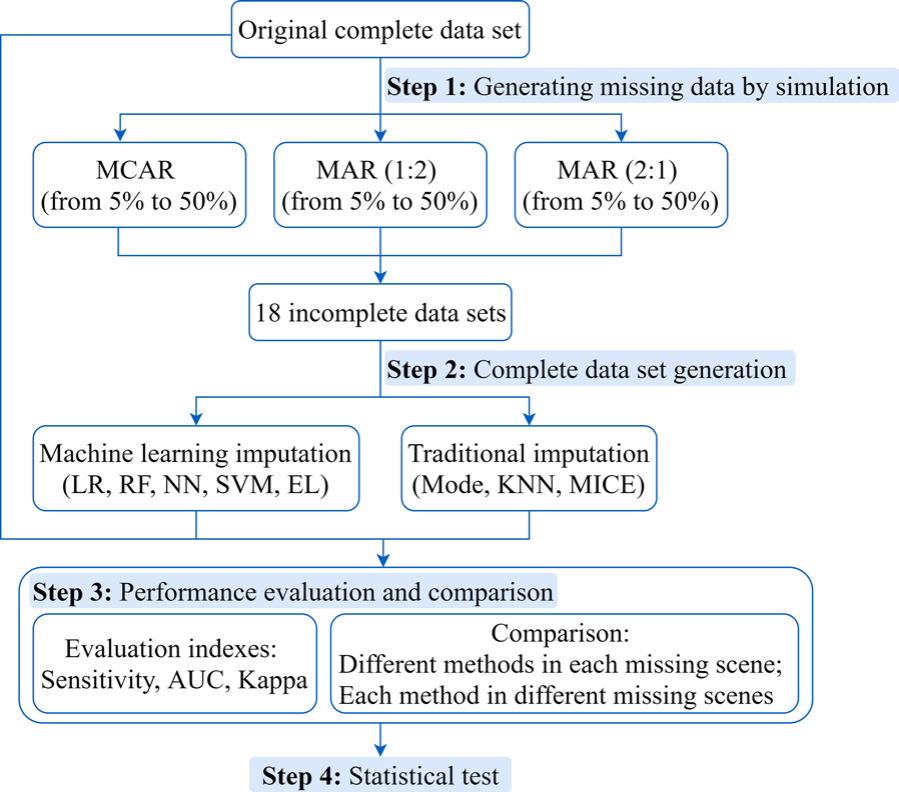
Experimental design. MAR (1:2): MAR (the ratio of missing proportion 1:2); MAR (2:1): MAR (the ratio of missing proportion 2:1)

Figure 1 showed the experimental design. The whole process consisted of four main steps: generating missing data by simulation, complete data set generation, performance evaluation and comparison, and statistical test.

#### Generating missing data by simulation

Missing mechanism, missing mode, missing proportion, data type of missing and requirements of processing method itself have impacts on the processing effect of missing data. It was comprehensively considered that this study created the corresponding missing scenario by setting the data missing mechanism of target variables, the proportion of missing and the ratio of missing proportion of each group. The target missing variable was set as the volume of supratentorial hemorrhage. The simulated missing data sets of different missing scenarios were artificially generated on the basis of the complete data.

According to the definition of Rubin DB [19], the data missing mechanism represents the relationship between missing of the target variable and other variables (including observed variables and unobserved variables) in the data set, which explains the reason for data missing. Particularly, it includes the missing completely at random (MCAR, the target variable independent of the observed and unobserved variables), missing at random (MAR, the target variable related to the observed variables) and missing not at random (MNAR, the target variable related to the unobserved variables). Because it is still difficult to simulate the MNAR mechanism, we set the missing mechanism as MCAR and MAR.

In the setting of MCAR mechanism, missing values were randomly generated. In the setting of MAR mechanism, we set the observed variable related to the target variable as the discharge situation. Specifically, the data set was split into two subsets according to the discharge situation, namely, the *failure* group and *success* group. We set the ratio of missing proportion of those two groups to 1:2 and 2:1, and controlled the total missing proportion of the two groups to the set proportion. In this way, the MAR (the ratio of missing proportion 1:2) and MAR (the ratio of missing proportion 2:1) mechanisms were formed to compare the effects of the ratio of missing proportion of each group on missing data processing methods under MAR mechanism.

Then, according to the possible missing situation in previous studies, the proportion of missing was set into six categories: 5%, 10%, 15%, 20%, 30% and 50% respectively, Finally, a total of 18 incomplete data sets corresponding to missing scenarios were generated by simulation.

#### Complete data set generation

Missing data processing techniques were applied to generate complete data sets by imputing missing values of the incomplete data sets of the previous step.

At present, there are three kinds of ideas in missing data processing, namely, deleting cases with missing values, weighting adjustment methods and missing values imputation. The imputation method is the mainstream of missing values processing. In view of the attention of missing data processing methods at present and comparing machine learning with traditional imputation, this study chose mode imputation (Mode) and KNN as the representatives of the traditional single imputation methods, multiple imputation by chained equations (MICE) as the representative of the traditional multiple imputation methods, and logistic regression (LR), RF, NN, support vector machine (SVM) and EL as the representatives of the machine learning imputation techniques.

##### Machine learning imputation

The missing data imputation methods based on machine learning usually use modeling to mine the effective information in the incomplete data, so as to reasonably infer the imputation values. The overall imputation idea of the following machine learning algorithms used in this study is to take the complete samples in the incomplete data set as the training set to establish the prediction model, and estimate the missing values according to the trained prediction model.

LR is one of the most commonly used and classic classification methods in machine learning [20]. It belongs to nonlinear regression, and is a multiple regression analysis method to study the relationship between the dependent variable with two or more classifications and some influencing factors. Because of its simplicity, easy implementation and maturity, it is widely used in classification problems.

RF proposed by Breiman L in 2001 is a derivative of ensemble learning Bagging algorithm [21]. The algorithm idea is as follows: ① the original data set is *N*, *m* samples are randomly sampled by Bootstrap method to form a training set which is repeated *B* times to obtain *B* training sets, and build *B* basic decision tree models. ② *p* features are randomly selected from all features, and then the best feature is selected from the *p* features according to the information gain for segmentation. ③ Each decision tree is split until the training samples of all nodes belong to the same class, and pruning is not needed in the whole process. ④ Generated *B* decision trees form a RF. This method not only pays attention to the performance of single decision tree classifier, but also reduces the correlation between each decision tree, improves the performance of combined classifier and increases the robustness of the algorithm to noise.

NN is a complex network system, in which neurons are connected with each other, and information is processed in parallel and converted nonlinearly by simulating the way of human brain nerve processing information. This study adopted the widely used back propagation neural network proposed by Rumelhart DE et al. in 1986 [22], which is a multilayer feedforward neural network trained by error back propagation algorithm. Back propagation neural network can learn and store a large number of input–output pattern mappings without revealing the mathematical equations describing the mappings in advance. Its learning rule is to use the steepest descent method to constantly adjust the weights and thresholds of NN through back propagation, to minimize the sum of squares of errors of NN. The most common three-layer back propagation neural network model was used in this study, including an input layer, a hidden layer and an output layer.

SVM was proposed by Vapnik V et al. [23]. It is designed for binary classification task, which can map linearly inseparable data to higher dimensional space and find a partition hyperplane with the largest interval in sample space based on training set to obtain decision function. By maximizing the margin between the two classes and minimizing the misclassification error, the samples of different classes are separated.

**Fig. 2:**
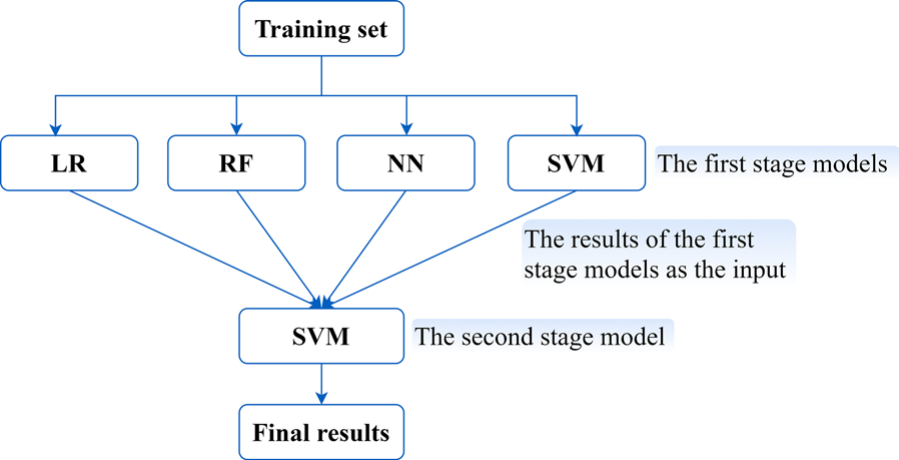
Stacking ensemble learning algorithm framework

EL accomplishes the learning task by constructing and combining multiple learners, and often obtains better generalization performance than a single learner [24]. This study adopted the Stacking algorithm proposed by Wolpert DH in 1992, also known as Stacked Generalization [25]. Stacking combines multiple classification methods into a single model, which takes advantages of different machine learning methods and thus improves the accuracy of prediction. For stacking, it has two-stage learning model. The original data set is used to train the first stage models, which include multiple different classification methods. The second stage model is trained to combine the prediction results from first stage models to obtain the final results. In this study, LR, RF, back propagation NN and SVM with radial basis function were used as the first stage models. For the second stage model, SVM with radial basis function was chosen to learn the relationships from the first stage models automatically. The algorithm framework was shown in Fig. 2.

According to the classification performance (Area Under Curve (AUC)) of ten-fold cross validation, the hyperparameters for each model and each incomplete data set simulated were tuned and the optimal configuration was selected using the Grid Search method. For example, Table 1 showed the optimal hyperparameter configuration of machine learning imputation techniques in the MAR (the ratio of missing proportion 1:2) mechanism scenario with a missing proportion of 5%.

**Table 1:** The optimal hyperparameter configuration of machine learning imputation techniques (under the MAR (the ratio of missing proportion 1:2) mechanism scenario with a missing proportion of 5%)

Methods	Packages	Hyperparameters to be tuned	Hyperparameters ranges	Optimal configuration
LR	–	–	–	–
RF	randomForest	mtry: number of randomly selected predictors	mtry = {1:8}	mtry = 4
NN	nnet	size: numbers of hidden units, decay: weight decay	size = {1:24}, decay = {0, 0.1, 0.01, 5e-4}	size = 4, decay = 0.1
SVM	Kernlab	sigma: Sigma^*^, C: cost	Kernel = Radial Basis Function Kernel, C = {0.25, 0.50, 1, 2, 4, 8, 16, 32}	Kernel = Radial Basis Function Kernel, C = 0.25
EL	kernlab, caret, caretEnsemble	sigma: Sigma^*^, C: cost	Kernel = Radial Basis Function Kernel, C = {0.25, 0.50, 1, 2, 4, 8, 16, 32}	Kernel = Radial Basis Function Kernel, C = 0.25

–: the parameter tuning is not required; ^*^: optimal configuration is automatically tuned

##### Traditional imputation

Mode is one of the simplest methods to impute missing value, which is to impute missing value with the mode of not missing value of each variable [26]. It is generally used for non-numerical variables.

KNN was first proposed by Cover T and Hart P in 1967 [27]. KNN realizes the imputation of missing values by mining the similarity between samples, which is to identify neighboring points by distance measurement, and then estimate missing value by using the complete values of neighboring points. Specifically, we can calculate the distance between a missing value and other complete values, find its *k* (*k* = 10) nearest distance data by using the defined function of distance between measured data (Euclidean distance), and then use the median of these *k* data to impute this missing value.

MICE is essentially a series of regression models, originally proposed by Boshuizen HC and Knook DL [28]. The missing values of each variable will be predicted according to other variables in the data, and repeated before the estimated value fully converges. At the same time, the whole process will be repeated *m* times, that is, after *m* times modeling and analysis, *m* different estimated values are generated for each missing value to form *m* complete data sets, and finally these *m* results are integrated according to certain rules to form the final missing value imputation result. This study adopted predictive mean matching with iterated 50 times to impute missing data 20 times repeatedly, and the average results of 20 times were integrated as the final imputation values.

#### Performance evaluation and comparison

In order to evaluate the impact of clinical decision-making results, the logistic regression models were constructed to evaluate the performance of missing data processing techniques. The discharge situation (*failure* (n = 261) = 1, *success* (n = 1207) = 0) as dependent variable and the other variables as independent variables, using the medical records of patients to assess their discharge. The imputation effects of missing data processing methods were evaluated by calculating the sensitivity, AUC and Kappa values of the models, which all ranged between 0 (the worst) and 1 (the best). The evaluation metrics values of original complete data were used as references.

The sensitivity reflects the extent to which the model can cover the concerned categories, that is, the proportion of patients correctly classified whose discharge situation are failure. It was calculated as shown in formula (1), where *TP* and *FN* denote true positives and false negatives, respectively.

1 $$Sensitivity = \frac{TP}{{TP + FN}}.$$

Because clinical decision making such as discharge assessment requires the prediction model to have high sensitivity, that is, to predict the failure of discharge as much as possible to avoid serious consequences in this study, specificity was not regarded as a separate metric for evaluation, and AUC was used to comprehensively reflect the accuracy combining sensitivity and specificity. The AUC can be acquired by calculating the area under the Receiver operating characteristic (ROC) curve plotting the true positive rate (sensitivity) against the false positive rate (1-specificity) over a range of cut-off values, which represents a trade-off between sensitivity and specificity.

The AUC represents accuracy, while the Kappa represents reliability, which is used to assess the consistency between the model results and the actual results. The Kappa was calculated as shown in formula (2), where *p*_*o*_ and *p*_*e*_ are the observed and expected by chance alone proportions of agreement, respectively.

2 $$Kappa = \frac{{p_{o} - p_{e} }}{{1 - p_{e} }}.$$

This study compared the performance of imputation techniques from two aspects: processing effects of different methods in each missing scenario and each method in different missing scenarios.

#### Statistical test

In order to evaluate whether the observed performance differences between different methods under different missing scenarios were statistically significant, the Wilcoxon signed-rank test was adopted. Due to multiple comparisons between multiple methods, the false discovery rate (FDR) method was used to adjust the *P* values. The statistical test level was 0.05.

In this study, R 4.0.1 software was used for data analysis. The packages used by traditional imputation included *DMwR2* and *mice*, while packages used by machine learning imputation were shown in Table 1.

## Results

### Data set description

Table 2 described the distribution of variables and corresponding categories of this study in the two groups of the discharge situation *failure* and *success*.

**Table 2:** Description of the data set

Variables	Categories	Discharge situation	
		Success (n = 1207)	Failure (n = 261)
Age	< 55	249 (20.6%)	37 (14.2%)
	55–64	265 (22.0%)	51 (19.5%)
	65–74	391 (32.4%)	86 (33.0%)
	75–84	246 (20.4%)	60 (23.0%)
	> 84	56 (4.6%)	27 (10.3%)
Gender	Male	688 (57.0%)	163 (62.5%)
	Female	519 (43.0%)	98 (37.5%)
More than two times of in-hospital	No	1194 (98.9%)	251 (96.2%)
	Yes	13 (1.1%)	10 (3.8%)
Deep coma	No	1190 (98.6%)	130 (49.8%)
	Yes	17 (1.4%)	131 (50.2%)
Diagnostic location	Deep	1081 (89.6%)	220 (84.3%)
	Superficial	126 (10.4%)	41 (15.7%)
Supratentorial hemorrhage volume	< 30 ml	1032 (85.5%)	128 (49.0%)
	≥ 30 ml	175 (14.5%)	133 (51.0%)
Operation	No	1045 (86.6%)	203 (77.8%)
	Yes	162 (13.4%)	58 (22.2%)
Co-infection	No	802 (66.4%)	138 (52.9%)
	Yes	405 (33.6%)	123 (47.1%)

### Analysis results of original complete data set fitting model

The sensitivity, AUC and Kappa values of the model fitted by the original complete data set in this study were 0.874, 0.914 and 0.558 as shown in Table 3, which can be used as reference for performance evaluation of missing data processing methods.

**Table 3:** Evaluation of logistic regression model fitting with original complete data set

	Sensitivity	AUC	Kappa
Original complete data set	0.874	0.914	0.558

### The performance comparisons of different methods in each missing scenario

#### Results in MCAR mechanism scenario

**Fig. 3:**
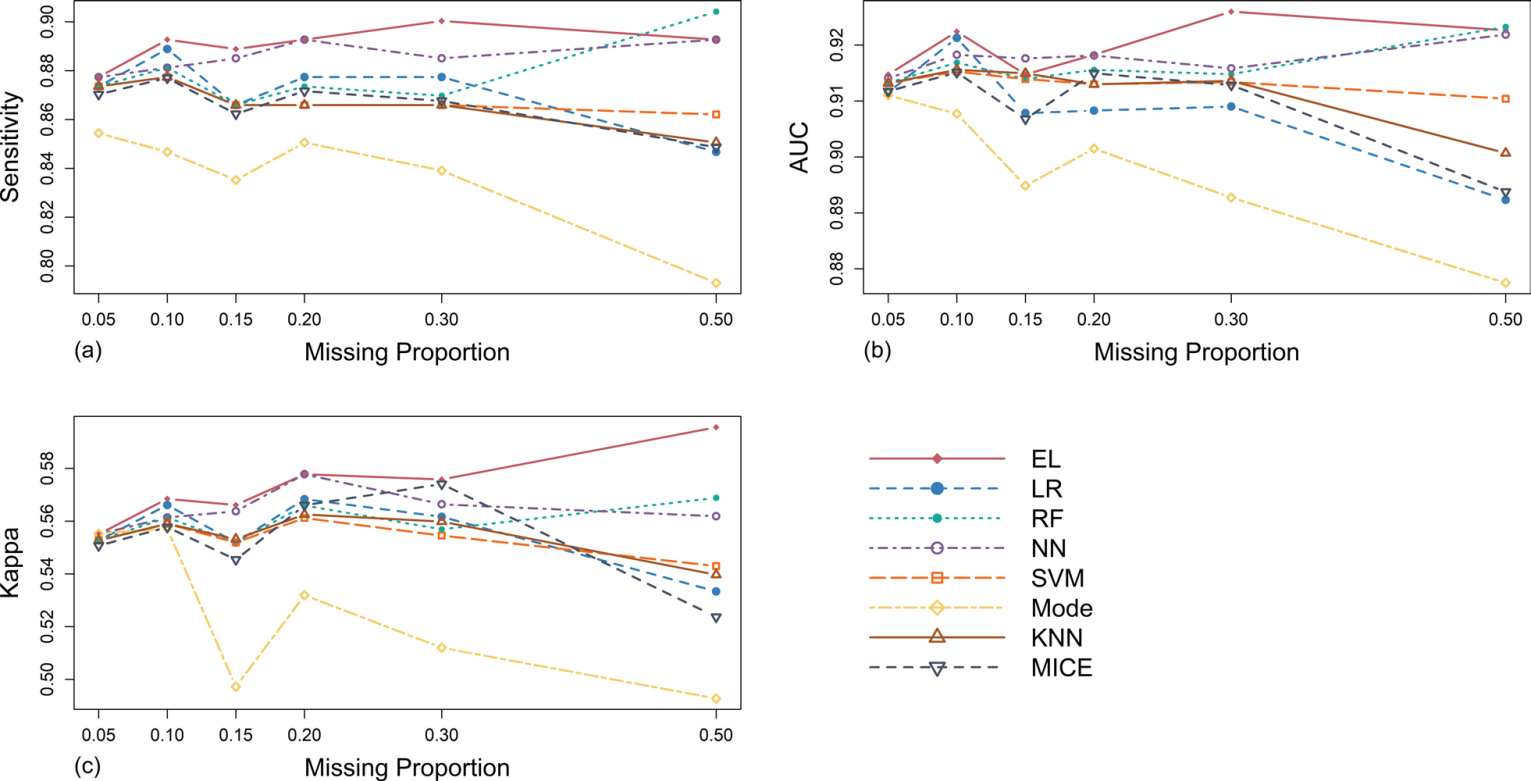
Simulation evaluation results of the sensitivity (**a**), AUC (**b**) and Kappa (**c**) values of each method under MCAR mechanism

As illustrated in Table 4 and Fig. 3, under the MCAR mechanism, in terms of sensitivity, the sensitivity values of Mode were the lowest among all methods under any missing proportion studied, and never reached that of the original complete data set. Meanwhile, with the gradual increase of missing proportion, it showed a downward trend as a whole. The sensitivity values and these overall change trends of KNN, MICE and SVM were similar under each missing proportion, and when the missing proportion was between 15 and 50%, they were lower than those of the original complete data set. The performance of RF was similar to that of the above three methods when missing proportion was lower than 30%. However, with the increase of missing proportion to 40% and 50%, the sensitivity values of RF increased obviously and significantly exceeded that of the original complete data set. The performance of NN was relatively stable, and its sensitivity values under any missing proportion studied were higher than that of the original complete data set. In comparison, EL had the best performance. Except that the sensitivity was slightly lower than that of RF when the missing proportion was around 50%, its performance was the best among the eight methods under the other missing proportions studied.

**Table 4:** Evaluation results of different processing methods in different scenarios of MCAR mechanism

Evaluation metrics	Missing proportions	Machine learning methods					Traditional methods		
		LR	RF	NN	SVM	EL	Mode	KNN	MICE
Sensitivity	0.05	0.874	0.874	0.877	0.874	0.877	0.854	0.874	0.870
	0.10	0.889	0.881	0.881	0.877	0.893	0.847	0.877	0.877
	0.15	0.866	0.866	0.885	0.866	0.889	0.835	0.866	0.862
	0.20	0.877	0.874	0.893	0.866	0.893	0.851	0.866	0.872
	0.30	0.877	0.870	0.885	0.866	0.900	0.839	0.866	0.868
	0.50	0.847	0.904	0.893	0.862	0.893	0.793	0.851	0.849
	Average	0.872	0.878	0.886	0.869	0.891	0.837	0.867	0.866
AUC	0.05	0.912	0.913	0.914	0.913	0.915	0.911	0.913	0.912
	0.10	0.921	0.917	0.918	0.915	0.922	0.908	0.916	0.915
	0.15	0.908	0.914	0.918	0.914	0.915	0.895	0.915	0.907
	0.20	0.908	0.916	0.918	0.913	0.918	0.901	0.913	0.915
	0.30	0.909	0.915	0.916	0.913	0.926	0.893	0.914	0.913
	0.50	0.892	0.923	0.922	0.910	0.923	0.877	0.901	0.894
	Average	0.908	0.916	0.918	0.913	0.920	0.898	0.912	0.909
Kappa	0.05	0.553	0.553	0.555	0.553	0.555	0.555	0.553	0.551
	0.10	0.566	0.561	0.561	0.559	0.568	0.557	0.559	0.558
	0.15	0.552	0.552	0.564	0.552	0.566	0.497	0.553	0.545
	0.20	0.568	0.566	0.578	0.561	0.578	0.532	0.563	0.566
	0.30	0.562	0.557	0.566	0.555	0.576	0.512	0.560	0.574
	0.50	0.533	0.569	0.562	0.543	0.596	0.493	0.540	0.524
	Average	0.556	0.560	0.564	0.554	0.573	0.524	0.555	0.553

The AUC trend of traditional imputation methods and SVM was similar to their sensitivity performance. The AUC value of LR was slightly higher than that of original complete data set when missing proportion was 10%, but lower under other missing proportions studied, and showed a downward trend as missing proportion increased gradually. Similarly, the performance of EL was the best on the whole.

In terms of the Kappa value, the performance of EL was relatively the best. However, Mode was obviously inferior to other methods and showed an obvious downward trend with the increase of missing proportion from 20%.

#### Results in MAR (the ratio of missing proportion 1:2) mechanism scenario

**Fig. 4:**
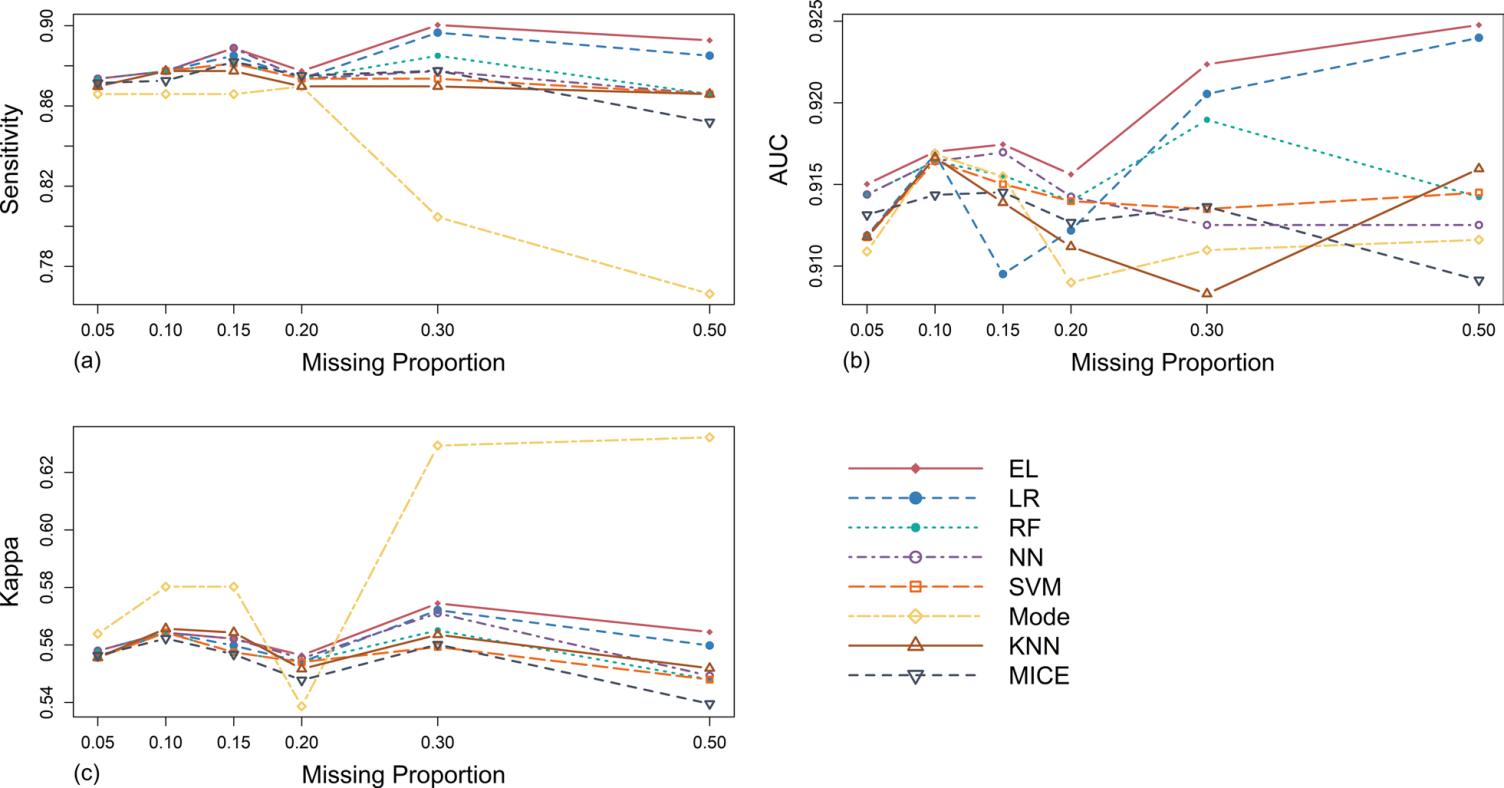
Simulation evaluation results of sensitivity (**a**), AUC (**b**) and Kappa (**c**) values of each method under MAR (the ratio of missing proportion 1:2) mechanism

From Table 5 and Fig. 4, in terms of sensitivity, under the low missing proportions (5%-20%), the performance of processing methods was similar, while the sensitivity of Mode was relatively low. Under the medium and high missing proportions (20%-50%), EL performed best, followed by LR. When the missing proportion was greater than 40%, the sensitivity of RF gradually decreased to lower than that of the original complete data set. Similarly, the sensitivity of Mode was obviously lower than other methods, and showed a cliff-like downward trend with the increase of missing proportion.

**Table 5:** Evaluation results of different processing methods under different scenarios of MAR (the ratio of missing proportion 1:2) mechanism

Evaluation metrics	Missing proportions	Machine learning methods					Traditional methods		
		LR	RF	NN	SVM	EL	Mode	KNN	MICE
sensitivity	0.05	0.870	0.874	0.874	0.870	0.874	0.866	0.870	0.871
	0.10	0.877	0.877	0.877	0.877	0.877	0.866	0.877	0.873
	0.15	0.885	0.881	0.889	0.881	0.889	0.866	0.877	0.882
	0.20	0.874	0.874	0.874	0.874	0.877	0.870	0.870	0.875
	0.30	0.897	0.885	0.877	0.874	0.900	0.805	0.870	0.878
	0.50	0.885	0.866	0.866	0.866	0.893	0.766	0.866	0.852
	Average	0.881	0.876	0.876	0.874	0.885	0.840	0.872	0.872
AUC	0.05	0.912	0.914	0.914	0.912	0.915	0.911	0.912	0.913
	0.10	0.917	0.916	0.916	0.916	0.917	0.917	0.917	0.914
	0.15	0.910	0.916	0.917	0.915	0.917	0.915	0.914	0.915
	0.20	0.912	0.914	0.914	0.914	0.916	0.909	0.911	0.913
	0.30	0.921	0.919	0.913	0.913	0.922	0.911	0.908	0.914
	0.50	0.924	0.914	0.913	0.915	0.925	0.912	0.916	0.909
	Average	0.916	0.916	0.915	0.914	0.919	0.913	0.913	0.913
Kappa	0.05	0.556	0.558	0.558	0.556	0.558	0.564	0.556	0.556
	0.10	0.564	0.564	0.564	0.564	0.564	0.580	0.566	0.562
	0.15	0.560	0.557	0.562	0.557	0.562	0.580	0.564	0.557
	0.20	0.554	0.554	0.555	0.554	0.556	0.539	0.552	0.548
	0.30	0.572	0.565	0.571	0.559	0.574	0.629	0.564	0.560
	0.50	0.560	0.548	0.549	0.548	0.564	0.632	0.552	0.540
	Average	0.561	0.558	0.560	0.556	0.563	0.587	0.559	0.554

As far as AUC was concerned, on the whole, the performance of EL was relatively the best, and always higher than that of the original complete data set under any missing proportion studied. Under the low missing proportions, the AUC values of NN were close to that of EL. Under the medium and high missing proportions, LR was closer to EL and showed an obvious upward trend.

As for Kappa, on the whole, Mode was particularly outstanding except that the missing proportion was about 20%. Under the medium and high missing proportions, EL had a slight advantage.

#### Results in MAR (the ratio of missing proportion 2:1) mechanism scenario

**Fig. 5:**
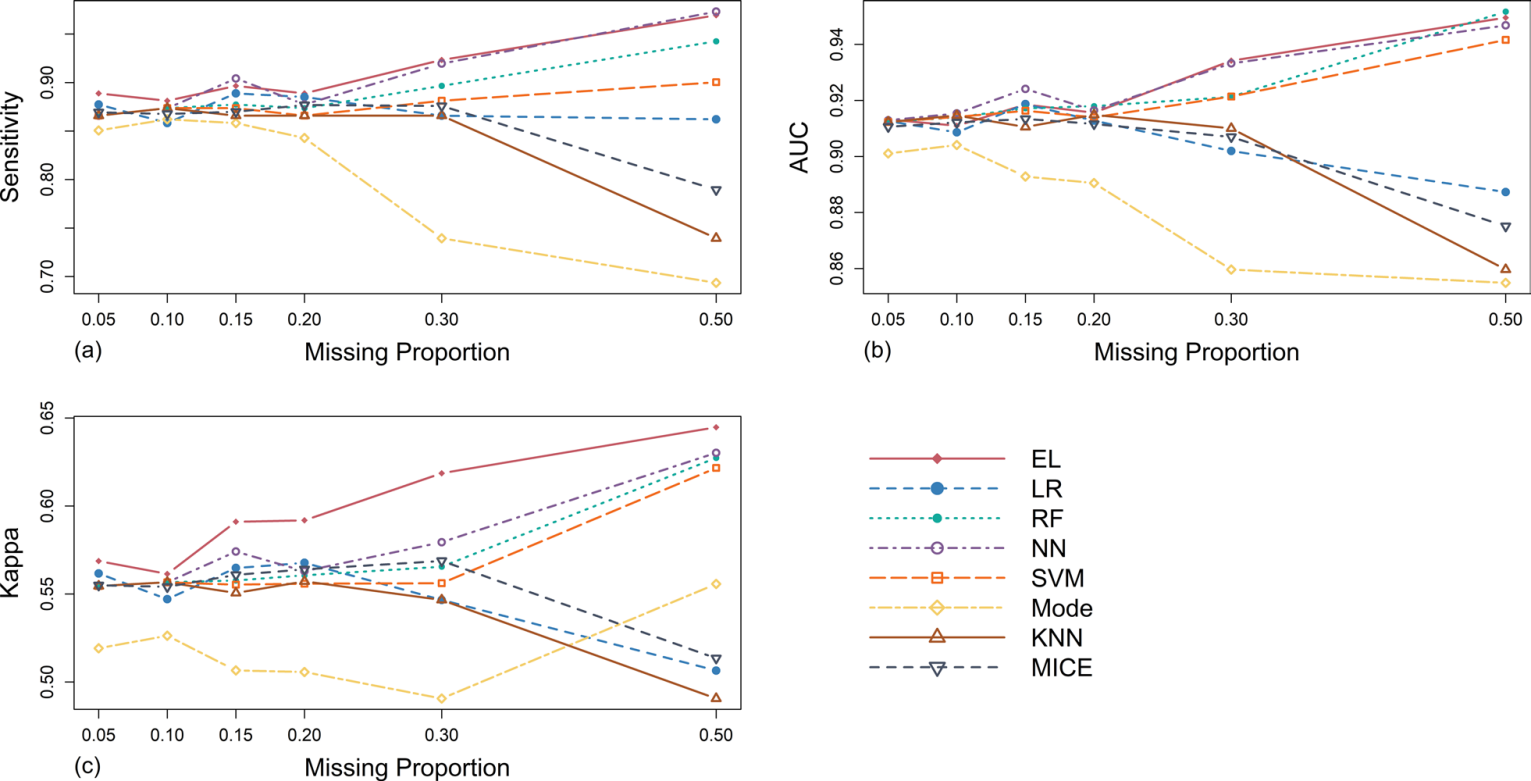
Simulation evaluation results of sensitivity (**a**), AUC (**b**) and Kappa (**c**) values of each method under MAR (the ratio of missing proportion 2:1) mechanism

As shown in Table 6 and Fig. 5, in terms of sensitivity, under the low missing proportions, the performance of processing methods was relatively close, while Mode was slightly inferior. Under the medium and high missing proportions, firstly, EL and NN were comparable and obviously superior to other methods. Secondly, the sensitivity values of RF and SVM were higher than that of the original complete data set and showed an upward trend with the increase of missing proportion. KNN and MICE showed an obvious downward trend under high missing proportions. However, Mode showed a cliff-like decline trend in the medium and high missing proportions, and its sensitivity values were at the lowest position.

**Table 6:** Evaluation results of different processing methods under different scenarios of MAR (the ratio of missing proportion 2:1) mechanism

Evaluation metrics	Missing proportions	Machine learning methods					Traditional methods		
		LR	RF	NN	SVM	EL	Mode	KNN	MICE
Sensitivity	0.05	0.877	0.866	0.866	0.866	0.889	0.851	0.866	0.869
	0.10	0.858	0.874	0.874	0.874	0.881	0.862	0.874	0.868
	0.15	0.889	0.877	0.904	0.874	0.897	0.858	0.866	0.870
	0.20	0.885	0.874	0.877	0.866	0.889	0.843	0.866	0.877
	0.30	0.866	0.897	0.920	0.881	0.923	0.739	0.866	0.876
	0.50	0.862	0.943	0.973	0.900	0.969	0.693	0.739	0.789
	Average	0.873	0.889	0.902	0.877	0.908	0.808	0.846	0.858
AUC	0.05	0.913	0.912	0.913	0.912	0.913	0.901	0.912	0.911
	0.10	0.909	0.914	0.915	0.914	0.911	0.904	0.915	0.912
	0.15	0.919	0.917	0.924	0.916	0.919	0.893	0.911	0.913
	0.20	0.913	0.918	0.916	0.914	0.916	0.891	0.915	0.912
	0.30	0.902	0.921	0.933	0.921	0.934	0.860	0.910	0.907
	0.50	0.887	0.952	0.947	0.942	0.950	0.855	0.860	0.875
	Average	0.907	0.922	0.925	0.920	0.924	0.884	0.904	0.905
Kappa	0.05	0.562	0.555	0.555	0.555	0.569	0.519	0.555	0.555
	0.10	0.547	0.557	0.557	0.557	0.561	0.526	0.557	0.554
	0.15	0.565	0.558	0.574	0.555	0.591	0.507	0.551	0.561
	0.20	0.568	0.561	0.563	0.556	0.592	0.506	0.557	0.564
	0.30	0.547	0.566	0.579	0.556	0.619	0.491	0.547	0.569
	0.50	0.507	0.627	0.630	0.622	0.645	0.556	0.491	0.514
	Average	0.549	0.571	0.576	0.567	0.596	0.518	0.543	0.553

As for AUC, the performance of Mode has never reached that of the original complete data set. Under the low missing proportions, NN had a slight advantage. Under the medium missing proportions, EL and NN were comparable. Under the high missing proportions, EL was slightly better than NN. In addition, under the medium and high missing proportions, RF and SVM showed an upward trend with the increase of missing proportion, while LR and the other two traditional imputation methods had a downward trend and gradually deviated from the reference values.

As far as Kappa value was concerned, the performance of EL was the best, and always better than that of original complete data set under any missing proportion studied. However, the performance of Mode was just the opposite. In addition, under the low and middle missing proportions, the Kappa values of the other six methods were relatively close and fluctuated around the level of original complete data set. When the missing proportion increased, NN, RF and SVM showed an upward trend, while LR and other two traditional imputation methods had an obvious downward trend.

### The performance comparisons of each method in different missing scenarios

**Fig. 6:**
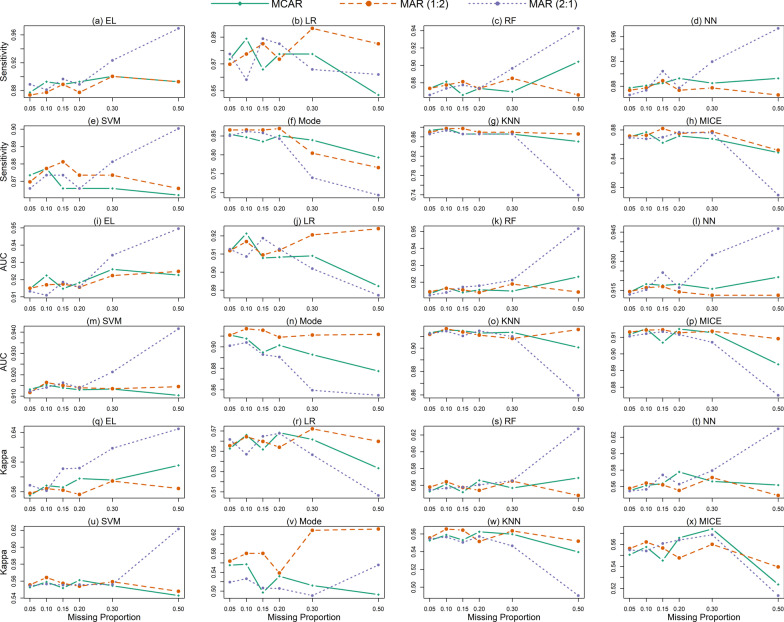
Comparison of processing effects of each method in different missing scenarios. **a** to **h**: the sensitivity comparison of different missing scenarios of EL, LR, RF, NN, SVM, Mode, KNN and MICE; **i** to **p**: AUC comparison; **q** to **x**: Kappa comparison; MAR (1:2): the MAR (the ratio of missing proportion 1:2) mechanism; MAR (2:1): the MAR (the ratio of missing proportion 2:1) mechanism

Figure 6 revealed the imputation performance difference of the same processing method under different missing mechanisms, which was more obvious when there was a high proportion of missing. Under the high missing proportions, EL, RF, NN and SVM had the best performance under the MAR (the ratio of missing proportion 2:1) mechanism. LR, KNN and MICE were more suitable for MAR (the ratio of missing proportion 1:2) mechanism. The sensitivity of Mode was higher under the MCAR mechanism, while the AUC and Kappa values were more prominent under the MAR (the ratio of missing proportion 1:2) mechanism.

### Results of the statistical test between EL and other methods

From the above descriptive and visual results, EL showed good performance compared with other methods. In order to evaluate whether the observed superior differences for EL with other methods were statistically significant, this study conducted the one-sided test on EL and other seven methods separately.

Table 7 confirmed the excellent performance of EL. Compared with Mode, KNN, MICE, LR and SVM, EL had relatively better performance under other missing mechanisms except that the differences between Kappa values under MAR (the ratio of missing proportion 1:2) mechanism and AUC values under MAR (the ratio of missing proportion 2:1) mechanism showed no statistical significance. In most cases of this study, the performance differences observed by descriptive analysis of EL better than NN were not statistically significant.

**Table 7:** The *P* values of statistical test between EL and other methods

Missing mechanisms	Evaluation metrics	*P* values	Machine learning methods				Traditional methods		
			LR	RF	NN	SVM	Mode	KNN	MICE
MCAR	Sensitivity	*p*.raw	0.018	0.047	0.091	0.016	0.016	0.016	0.016
		*p*.adj	0.025	0.055	0.091	0.025	0.025	0.025	0.025
	AUC	*p*.raw	0.016	0.029	0.139	0.018	0.016	0.030	0.018
		*p*.adj	0.031	0.034	0.139	0.031	0.031	0.034	0.031
	Kappa	*p*.raw	0.018	0.016	0.050	0.016	0.030	0.016	0.016
		*p*.adj	0.025	0.025	0.050	0.025	0.034	0.025	0.025
MAR (the ratio of missing proportion 1:2)	Sensitivity	*p*.raw	0.028	0.050	0.091	0.030	0.016	0.030	0.016
		*p*.adj	0.041	0.059	0.091	0.041	0.041	0.041	0.041
	AUC	*p*.raw	0.029	0.017	0.029	0.018	0.030	0.029	0.018
		*p*.adj	0.030	0.030	0.030	0.030	0.030	0.030	0.030
	Kappa	*p*.raw	0.024	0.050	0.091	0.029	0.953	0.086	0.016
		*p*.adj	0.068	0.088	0.106	0.068	0.953	0.106	0.068
MAR (the ratio of missing proportion 2:1)	Sensitivity	*p*.raw	0.016	0.018	0.172	0.017	0.016	0.018	0.016
		*p*.adj	0.021	0.021	0.172	0.021	0.021	0.021	0.021
	AUC	*p*.raw	0.050	0.584	0.819	0.086	0.016	0.071	0.031
		*p*.adj	0.117	0.681	0.819	0.120	0.109	0.120	0.109
	Kappa	*p*.raw	0.016	0.016	0.016	0.018	0.016	0.016	0.016
		*p*.adj	0.018	0.018	0.018	0.018	0.018	0.018	0.018

*p*.raw: the *p* value of Wilcoxon signed rank test;

*p*.adj: the *p* value adjusted by the FDR method based on *p*.raw

## Discussion

This study applied machine learning imputation techniques to deal with the missing of data in clinical decision making. The results showed that the accuracy of EL was imporved by combining the advantages of multiple single learners when dealing with missing data problem. Because EL was composed of multiple learners to solve the same problem, it can effectively alleviate the over-fitting problem of a single learner and improve the generalization ability to a certain extent. In addition, it can be seen that the performance of EL imputation results in different missing scenarios was generally higher, and there was no phenomenon of ups and downs. Thus, the stability of its imputation results was higher than that of a single learner.

Other single machine learning algorithms had different performances in different missing scenarios. Under the mechanism of MCAR and MAR (the ratio of missing proportion 2:1), the performance of NN was only inferior to EL. Firstly, NN needs large sample data to train and learn for building models. The sample size of this study can meet the requirement of NN to a large extent, which can ensure that NN can give full play to its advantages under the condition of large sample. Secondly, because of its strong adaptability, NN can adapt to the new environment in time by retraining when the conditions change slightly, so that its prediction performance is not sensitive to the missing data itself and the amount of missing data. At the same time, the NN also has certain fault tolerance, and the local errors of the network will not have a severe impact on the whole. Therefore, these characteristics of NN combined with the data conditions in this study make its imputation performance stand out. Under these two missing mechanisms, when the missing proportion was high, the imputation effect of RF was second only to the NN. Because of the characteristics of random selection of samples, random selection of features and the construction of multiple decision trees, RF is not easy to produce over fitting.

In addition, under MAR (the ratio of missing proportion 2:1) mechanism, when missing proportion was high, the performance of SVM was only inferior to that of EL, NN and RF. From the theoretical basis and characteristics of SVM itself, it is a machine learning method based on statistical theory, which is mainly suitable for limited samples and has excellent learning ability based on limited information in small samples. However, when the sample size is large, the separability of data and the accuracy of classification may decrease. Therefore, only under the missing scenario of MAR (the ratio of missing proportion 2:1) mechanism and high missing proportion set in this study, the advantages of SVM were slightly prominent. On the one hand, because of the high missing proportion, the available sample information was more limited. On the other hand, under the MAR (the ratio of missing proportion 2:1) mechanism, the missing proportion of the target variable in the *failure* group was twice as high as that in the *success* group, and the original sample size of the *failure* group was small, which made the missing degree of the target variable in *failure* group more serious and the available sample information less. So compared with other missing scenarios, SVM in this scenario was more prominent.

Under MAR (the ratio of missing proportion 1:2) mechanism, and the medium and high missing proportions, LR was second only to EL. It can be seen that the different ratios of missing proportions of groups will affect the imputation effect of missing data processing method. Compared with the ratio of missing proportion 2:1, the imputation method based on LR was more efficient when the ratio of missing proportion was 1:2.

On the whole, the performance of machine learning imputation was better than that of traditional imputation methods, especially in the case of a large proportion of missing. The performance of traditional imputation methods, KNN and MICE, had no obvious advantages and continued to weaken at high missing proportions, which was difficult to meet the requirement. As far as Mode was concerned, its implementation was very simple. However, it ignored the relationship between the value of missing and other variables, which made its imputation performance was not ideal in most missing scenarios compared with other methods. This finding was consistent with the conclusion of previous research [26]. However, this study also found that under MAR (the ratio of missing proportion 1:2) mechanism, when the missing proportion was greater than about 25%, the Kappa of Mode was much higher than that of other methods. This result was also reasonable. It was mainly affected by missing mechanism and the ratio of missing proportion of groups. With the gradual increase of the missing proportion, the missing of target variable in the *success* group would become more and more serious. At this time, for the imputation of missing values in the *success* group, it may be more reasonable to use the mode of non-missing values. The specificity would be higher, which led to the excellent relatively Kappa value performance of Mode in this missing scenario.

The main contribution of this study was to provide the methodological application mechanism and enhance the reliability of evidence for machine learning, especially EL, to better solve the problem of missing data clinically, providing methodological support for clinical decision making in presence of missing data. However, it still had several limitations in the setting of missing scenario. For example, considering the convenience of implementation and interpretation, only the monotonous missing mode was set. But the actual situation is often more complicated, so the practical value of machine learning imputation methods needs to be further explored and improved in the richer missing scenarios.

## Conclusions

The performances of missing data processing methods were different to a certain extent in different missing scenarios. On the whole, machine learning had better imputation performance than traditional methods, especially in scenarios with high missing proportions. Compared with single machine learning algorithms, the performance of EL was more prominent, followed by NN. Meanwhile, EL was most suitable for missing imputation under MAR (the ratio of missing proportion 2:1) mechanism, and its average sensitivity, AUC and kappa values reached 0.908, 0.924 and 0.596 respectively. At the same time, this study also revealed that the data missing mechanism, missing proportion and ratio of missing proportion of each group were essential factors to be considered when formulating missing data processing strategies. The findings of this study shed light on the development of missing data processing technology, and provided methodological support for clinical decision making in presence of incomplete data.

## Data Availability

The datasets analysed during the current study are not publicly available due to privacy but are available from the corresponding author on reasonable request.

## Abbreviations

EL: Ensemble learning
KNN: K-nearest neighbors
RF: Random forest
NN: Neural networks
MCAR: Missing completely at random
MAR: Missing at random
MNAR: Missing not at random
Mode: Mode imputation
MICE: Multiple imputation by chained equations
LR: Logistic regression
SVM: Support vector machine
AUC: Area under curve
ROC: Receiver operating characteristic
FDR: False discovery rate
